# Effects of different complementary therapies on chronic kidney disease-associated pruritus: a systematic review and network meta-analysis

**DOI:** 10.3389/fmed.2026.1860437

**Published:** 2026-07-22

**Authors:** Shu He, Wenxia Huang, Yan Zhou, Siyu Du

**Affiliations:** 1 West China School of Nursing, West China Hospital, Sichuan University, Chengdu, China; 2 General Practice Medical Center, West China Hospital, Sichuan University, Chengdu, China

**Keywords:** Bayesian, CKD-aP, complementary therapies, network meta-analysis, R software

## Abstract

**Background:**

Chronic kidney disease-associated pruritus is one of the most common concomitant symptoms in patients with end-stage renal disease. It leads to multiple adverse outcomes for patients. Complementary therapies are increasingly used and represent an effective alternative approach. However, the optimal intervention method has not yet been clearly established.

**Objective:**

To compare the efficacy and safety of different complementary therapies in alleviating symptoms of chronic kidney disease-associated pruritus.

**Methods:**

A systematic search was conducted in 12 databases and relevant medical websites in accordance with the PRISMA-NMA guidelines. Randomized controlled trials published in the database from database inception through December 2025 were included. The Cochrane risk-of-bias tool for randomized trials (RoB 2.0) was used to assess the quality of the included literature. High-risk-of-bias studies were excluded and subsequently re-included in the sensitivity analysis. Statistical analysis was performed using Bayesian methods with the “gemtc” package and other R packages. The quality of evidence was assessed using the CINeMA online platform.

**Results:**

A total of 29 studies involving 12 complementary and alternative approaches and 1,968 patients were included. The network meta-analysis showed that Acupuncture (SMD = −2.12, 95% CI = −4.17 to −0.13; SUCRA = 0.80), Aromatherapy (SMD = −2.05, 95% CI = −4.00 to −0.08; SUCRA = 0.78), Herbal Medicinal Bath (SMD = −1.69, 95% CI = −2.67 to −0.71; SUCRA = 0.72) and Combination therapy (SMD = −1.47, 95% CI = −2.53 to −0.43; SUCRA = 0.65) were superior to the control group in improving pruritus symptoms. However, when the 9 excluded high-risk-of-bias studies were re-included in a sensitivity analysis, the effects of Acupuncture and other therapies were attenuated and no longer statistically significant. Only Herbal Medicinal Bath maintained a statistically significant and stable effect (SMD = −2.40, 95% CI = −3.60 to −1.21; SUCRA = 0.82).

**Conclusion:**

Based on the efficacy and stability of the current evidence, Herbal Medicinal Bath appears to be the most reliable complementary therapy. Acupuncture shows potential but requires further confirmation through more high-quality, large-scale randomized controlled trials.

**Systematic review registration:**

https://www.crd.york.ac.uk/PROSPERO/view/CRD420261279443, identifier CRD420261279443.

## 1 Introduction

Recent surveys indicate that the global prevalence of chronic kidney disease (CKD) among adults is 788 million, with 19.951 million new cases. CKD has become the ninth leading cause of death worldwide, and this burden is projected to increase further by 2030 (1). Although refined treatment strategies and advances in dialysis techniques have prolonged patient survival, individuals with end-stage renal disease still experience a range of concomitant symptoms that severely impair quality of life and prognosis (2, 3). Pruritus is one of the most common and distressing symptoms. Studies report an overall prevalence of 52%, comprising mild (26%), moderate (22%), and severe (8%) cases (4, 5). Chronic kidney disease-associated pruritus (CKD-aP), also known as uremic pruritus (UP), is pruritus secondary to kidney disease that cannot be attributed to other causes. It typically presents as localized or generalized itching of varying intensity, with or without rash, and is often episodic with nocturnal exacerbations (6, 7). Mild CKD-aP increases the risk of skin lesions, bleeding, and infection, whereas severe cases can lead to depression and sleep disturbances. Over the long term, the cumulative burden of CKD-aP may worsen the patient’s illness experience and elevate mortality risk (8).

Current management of CKD-aP primarily includes kidney transplantation, dialysis, and pharmacotherapy. Kidney transplantation is the most effective option, but its accessibility is limited. Dialysis offers only modest benefit and is contingent upon equipment availability. Pharmacotherapy is associated with numerous challenges, including variable efficacy, the absence of standardized protocols, notable adverse effects, and substantial medication burden (9, 10). These limitations underscore the importance of identifying therapeutic approaches that are safe, effective, affordable, and accessible. Recent expert consensus recommends complementary therapies such as acupuncture, acupressure, ultraviolet phototherapy, and herbal medicine to alleviate CKD-aP, and a summary of the evidence further confirms that these therapies can effectively relieve pruritus with a favorable safety profile (11, 12). In recent years, complementary and alternative therapies have proliferated. However, most randomized controlled trials (RCTs) have used Usual Care as the comparator, and only a few have involved direct comparisons of complementary therapies. Previous meta-analyses have been largely confined to pooling studies of the same complementary therapy (13–16). Consequently, the available data are relatively limited and demonstrate inconsistent efficacy. The optimal intervention for CKD-aP, therefore, remains undetermined. A comprehensive evaluation of the efficacy and safety of different complementary therapies is thus of considerable importance for improving CKD-aP.

Network meta-analysis is an analytical approach that integrates both direct and indirect comparative evidence to evaluate the treatment effects of different interventions and to rank them accordingly (17). Therefore, this systematic review aims to compare the effects of various complementary therapies on chronic kidney disease-associated pruritus using the currently available evidence, thereby providing a reference for clinical decision-making and guiding future practice.

## 2 Methods

This study adhered to the PRISMA-NMA extension statement for reporting systematic reviews. The study protocol was registered in PROSPERO (Registration No. CRD420261279443).

### 2.1 Inclusion and exclusion criteria

Inclusion Criteria: ① Population: Participants were adults (≥18 years) with stage 5 chronic kidney disease who were undergoing dialysis and experiencing pruritus. There were no restrictions regarding dialysis modality, prior treatment regimens, pruritus type, or pruritus severity. ② Interventions: Included studies involved at least one complementary therapy, including Aromatherapy, Ultraviolet Phototherapy, Music Therapy, Herbal Medicinal Bath, Herbal Fumigation and Steam, Herbal Enema, Acupuncture, Moxibustion, Auricular Acupressure, Acupressure, Gua Sha, or Combination Therapy. ③ Comparators: Studies were required to include a control group, which could consist of Usual Care, Sham Treatment, or an alternative complementary therapy. ④ Outcomes: The primary outcomes of interest were pruritus severity and the incidence of adverse events. ⑤ Study Design: Randomized controlled trials (RCTs).

Exclusion Criteria: ① Full text or key data were unavailable. ② Duplicate or overlapping publications. ③ Studies published in languages other than English or Chinese. ④ Studies assessed as having a high risk of bias.

### 2.2 Search strategy

A systematic search was conducted using a combination of subject headings and free-text terms in both Chinese and English databases, including CNKI, Wanfang, VIP, SinoMed, PubMed, Cochrane Library, Embase via Ovid, Web of Science, ScienceDirect, CINAHL, ProQuest, and Scopus, as well as relevant medical websites such as ClinicalTrials.gov. Gray literature and the reference lists of included articles were also searched. The search covered the period from database inception through December 2025. We used the following Boolean operators in the search: (“chronic kidney disease” OR “uremia”) AND “pruritus” AND “randomized controlled trial” AND (“complementary therapy” OR “aromatherapy” OR “phototherapy” OR “music therapy” OR “external therapy” OR “herbal external washing” OR “Chinese herbal bath” OR “hydrotherapy” OR “steaming washing therapy” OR “moisten compress therapy” OR “herbal enema” OR “acupuncture moxibustion therapy” OR “acupoint therapy” OR “Scraping Therapy”). Synonyms were used flexibly throughout the search process to ensure comprehensive retrieval of the literature. The detailed search strategy is provided in Supplementary Material 1.

### 2.3 Study selection and data extraction

Study selection was managed using EndNote X9 software. Duplicate records were first removed. Two researchers independently screened all titles and abstracts, followed by a full-text review in accordance with the inclusion and exclusion criteria. Any disagreements were resolved through discussion or by consulting a third researcher. Data were extracted from the included studies using an Excel spreadsheet and cross-checked for accuracy. The extracted information included author names, publication year, country, mean age, sample size, intervention type, intervention protocol, treatment duration, outcome measurement tools, outcome indicators, and adverse events.

### 2.4 Quality assessment

Two researchers independently assessed the methodological quality of the included studies using the Cochrane risk-of-bias tool for randomized trials (RoB 2.0). Disagreements were resolved by consensus or through consultation with a third researcher. The tool covers five domains: (1) bias arising from the randomization process; (2) bias due to deviations from intended interventions; (3) bias due to missing outcome data; (4) bias in measurement of the outcome; and (5) bias in selection of the reported result. Each domain was evaluated for risk of bias, and the overall risk of bias for each study was categorized as “low risk,” “some concerns,” or “high risk” (18).

### 2.5 Statistical analysis

A Bayesian framework was adopted for this study. All analyses were performed using R version 4.4.1 and multiple packages within RStudio. All scales adopted a positive scoring system, with higher scores indicating greater pruritus severity and lower scores indicating symptom improvement. Effect size calculation: To eliminate measurement differences across different outcome measurement scales (pruritus severity), the mean change from baseline to endpoint and the corresponding standard deviation were used to calculate the summary effect size. Standardized mean differences (SMDs) were computed using the software, yielding difference values (diff) and standard errors (std.er). The data were then formatted for compatibility with the “gemtc” package in R (19, 20).

#### 2.5.1 Network plot

The network of included data was visualized using the “igraph” and “dplyr” packages in RStudio to illustrate direct and indirect comparisons among different interventions. Each node in the plot represents an intervention, with node size proportional to sample size. Connecting lines indicate direct comparisons between interventions in the included studies, with line thickness proportional to the number of studies reporting that comparison.

#### 2.5.2 Bayesian model construction

Bayesian network analysis was conducted using the “gemtc,” “rjags,” and “dmetar” packages in RStudio, employing the Monte Carlo Markov Chain (MCMC) method. Model parameters were set as follows: non-informative priors, four chains, 20,000 adaptation iterations, 50,000 total iterations, and a thinning interval of 1. Model convergence was assessed using the potential scale reduction factor (PSRF), with values closer to 1 indicating satisfactory convergence and stable posterior estimates (21). Effect sizes for pairwise comparisons were expressed as SMDs with 95% credible intervals (95% CI) and presented in league tables and forest plots. To evaluate the relative efficacy of interventions, surface under the cumulative ranking curve (SUCRA) values were calculated and visualized using grouped rank plots. SUCRA values approaching 1 indicate greater efficacy, whereas values approaching 0 indicate less favorable outcomes.

#### 2.5.3 Assessment of transitivity, heterogeneity, and inconsistency

The transitivity assumption was evaluated based on study characteristics. Statistical heterogeneity was assessed using τ^2^ and I^2^. Between-study variance τ^2^ was classified according to established thresholds: low (<0.04), low to moderate (0.04–0.16), moderate to high (0.16–0.36), and high (>0.36). The heterogeneity statistic I^2^ was categorized as none (0%), low (25%), moderate (50%), or high (75%) (22, 23). When heterogeneity was high, a random-effects model was selected, and meta-regression was performed to explore potential sources of heterogeneity by including outcome measurement tool, treatment duration, sample size, publication year, and mean age as covariates. Inconsistency between direct and indirect evidence was examined using both global and local approaches. Global inconsistency was tested by comparing the deviance information criterion (DIC) of the consistency model with that of the inconsistency model; a DIC difference of less than 5 indicates no significant global inconsistency (20). Local inconsistency was evaluated using the node-splitting method; *p*-values ≥ 0.05 indicated no significant inconsistency.

#### 2.5.4 Sensitivity analysis and publication bias

To assess the robustness of the findings, a leave-one-out sensitivity analysis was performed. Changes in network structure, effect estimates, between-group heterogeneity, and treatment rankings were examined after sequentially omitting individual studies. Results were considered unstable if omitting a study altered or disrupted the network structure, substantially shifted effect estimates, or markedly changed the ranking order (24, 25). To evaluate the impact of excluding high-risk-of-bias studies, we re-included the nine RCTs that had been initially excluded because of a high overall risk of bias. We then conducted a secondary sensitivity analysis to examine whether the effect sizes, direction of effects, intervention rankings, and heterogeneity parameters changed. Publication bias was visually evaluated using a comparison-adjusted funnel plot. The dashed line perpendicular to the horizontal axis represents the pooled effect estimate. Studies with larger sample sizes and higher precision are expected to appear near the top of the funnel, while smaller and less precise studies appear near the bottom. A roughly symmetric distribution suggests a low risk of publication bias (26).

### 2.6 Certainty of evidence assessment

The Confidence in Network Meta-Analysis (CINeMA) online tool was used to evaluate the certainty of evidence across six domains: within-study bias, reporting bias, indirectness, imprecision, heterogeneity, and inconsistency. Each domain was rigorously assessed, and the overall confidence in the evidence was rated accordingly based on the evaluation results.

## 3 Results

### 3.1 Study selection process and basic characteristics

**FIGURE 1:**
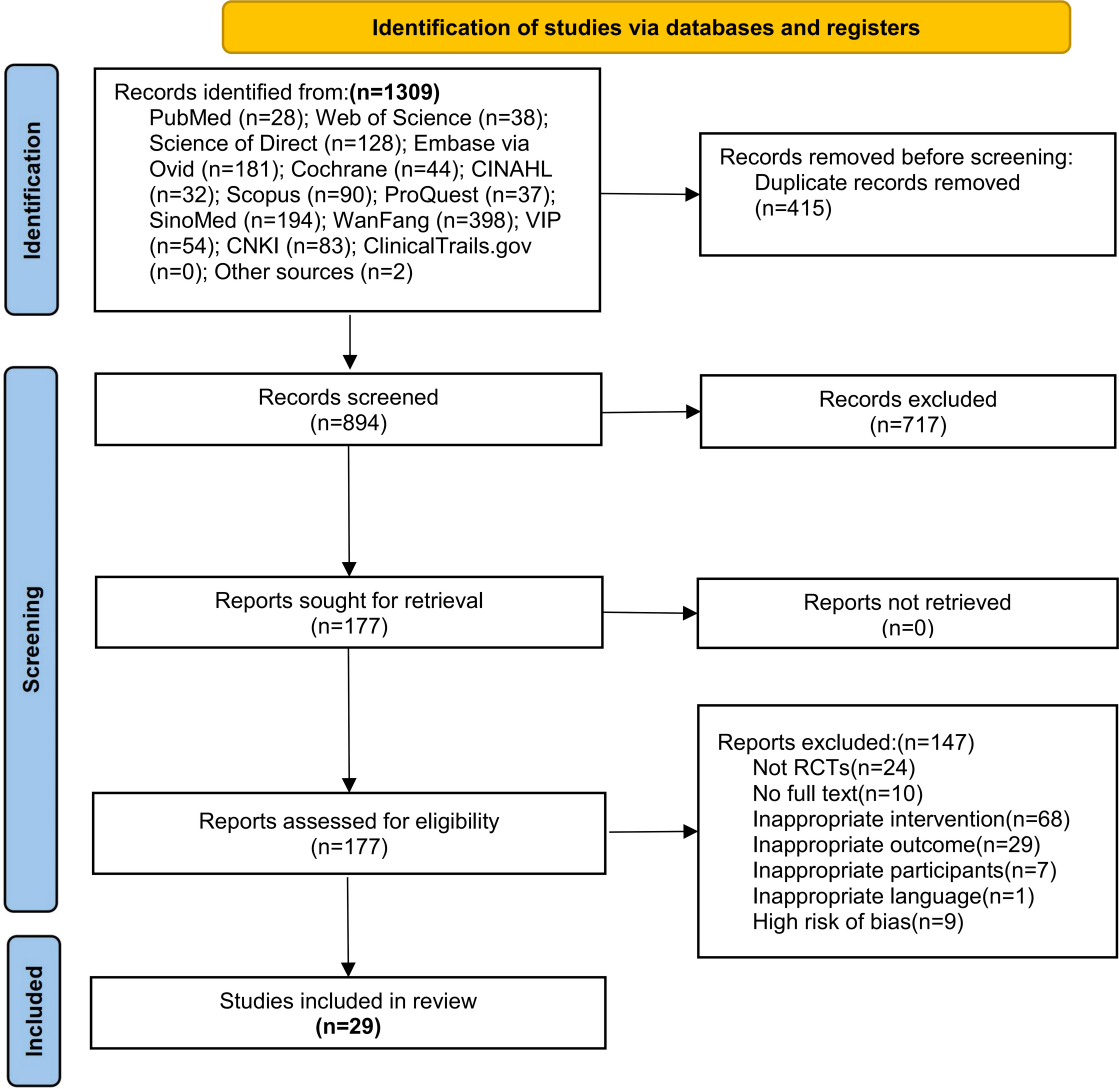
PRISMA flow diagram of the search process for studies.

A total of 1,309 records were initially retrieved through the search strategy. After removing 415 duplicate records, 717 records were excluded following title and abstract screening based on relevance to the study topic. Subsequently, full-text articles were assessed according to the inclusion and exclusion criteria, resulting in the exclusion of 148 studies. Ultimately, 29 studies were included in the final analysis. The detailed study selection process is illustrated in Figure 1.

Of the 29 included studies, 26 were two-arm parallel randomized controlled trials (27–52), and three were three-arm clinical trials (53–55). For network meta-analysis, only data from the selected intervention and control arms were extracted from the three-arm trials. The included studies comprised 1,968 participants, with mean ages ranging from 41.4 to 68.9 years. The studies were published between 2005 and 2025 and were conducted in four countries: China, Iran, Indonesia, and Italy. Intervention durations ranged from 2 to 12 weeks. Instruments used to assess pruritus severity included the Visual Analog Scale (VAS), the Modified Duo’s Pruritus Scale, the Dirk R. Kuypers Scale, the Four-Item Itch Questionnaire (FIIQ), the Chinese version of the 14-item Uremic Pruritus Scale, and the 5-D Itch Scale. Lower scores on these instruments indicate milder pruritus severity.

The included studies involved 12 distinct interventions. Four studies (28, 30–32) investigated Acupuncture; two studies each investigated Auricular Acupressure (51, 52), Herbal Fumigation and Steam (36, 37), Ultraviolet Phototherapy (33, 40), Gua Sha (47, 50), Acupressure (27, 53), and Aromatherapy (27, 54); one study each investigated Moxibustion (48), Herbal Enema (55), and Music Therapy (29). Six studies (34, 35, 37, 38, 44, 45) examined Combination Therapy, and seven studies (39–43, 46, 49) examined Herbal Medicinal Bath. Regarding control conditions, seven studies (28, 30–32, 52–54) used Sham Treatment as the comparator, 21 studies (29, 33–36, 38, 39, 41–51, 53–55) involved interventions added to Usual Care, and three studies (27, 37, 40) employed head-to-head comparisons between active interventions. The basic characteristics of the included studies are presented in Supplementary Material 2.

### 3.2 Quality assessment

**FIGURE 2:**
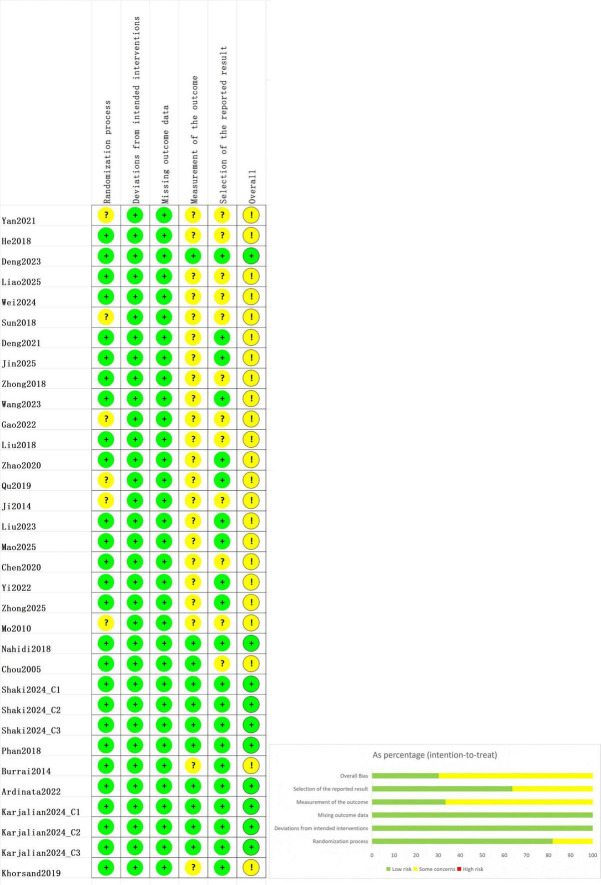
Risk of bias.

In accordance with the Cochrane guidelines, nine studies assessed as having an overall high risk of bias were excluded from the primary analysis to prioritize internal validity. Among the included studies, six met all quality criteria and were assessed as having a low overall risk of bias. The remaining 23 studies were rated as having some concerns. The primary limitations included insufficient description of random sequence generation and allocation concealment mechanisms, inadequate reporting of blinding procedures for personnel, participants, and outcome assessors, and a lack of information regarding selective outcome reporting. These limitations may introduce potential bias. A detailed risk-of-bias assessment is presented in Figure 2.

### 3.3 Pairwise meta-analysis and network meta-analysis

**FIGURE 3:**
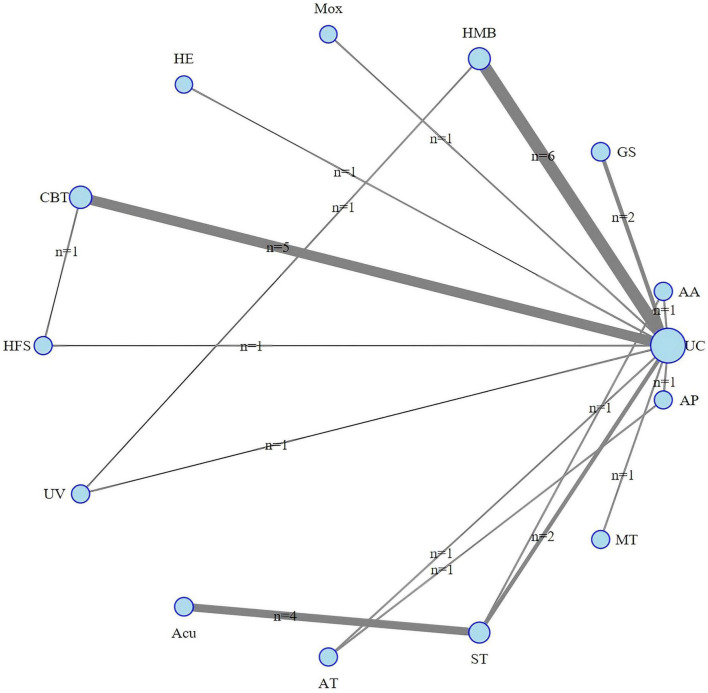
Network evidence graph. UC, Usual Care; ST, Sham Treatment; AA, Auricular Acupressure; Acu, Acupuncture; AT, Aromatherapy; HMB, Herbal Medicinal Bath; CBT, Combination Therapy; HFS, Herbal Fumigation and Steam; GS, Gua Sha; HE, Herbal Enema; Mox, Moxibustion; UV, Ultraviolet Phototherapy; MT, Music Therapy; AP, Acupressure.

The network structure and the number of studies examining interactions among therapies are shown in Figure 3. The evidence network is fully connected. Usual Care (UC) had the largest sample size, and its corresponding node is therefore the largest in the plot. All interventions are connected through a common comparator and form well-defined closed loops within the network.

Convergence diagnostics for the Bayesian model indicated that the potential scale reduction factor (PSRF) values for all intervention comparisons and model parameters stabilized at 1. Trace plots showed that the four Markov chains intermingled and stabilized within a horizontal band, with no discernible periodic or trend fluctuations. Posterior density plots displayed regular, unimodal, symmetric distributions with smooth shapes. Collectively, these findings confirm that the model converged well and that the posterior estimates were stable and reliable. Further details are provided in Supplementary Materials 3, 4.

**FIGURE 4:**
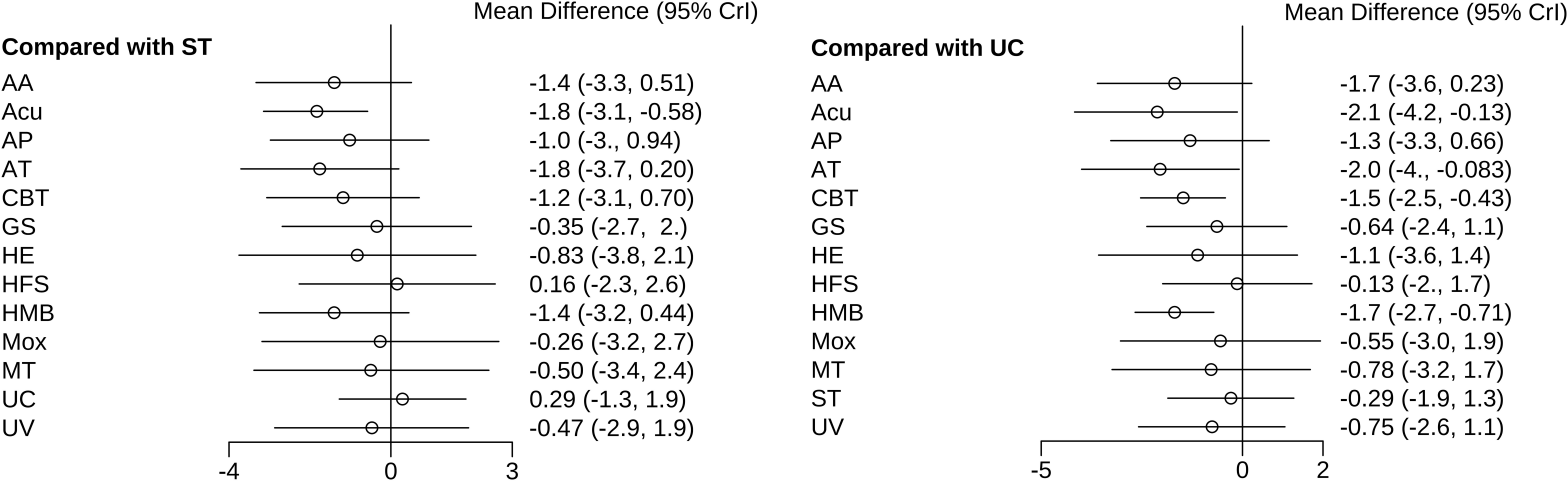
Forest plot for paired comparison.

**FIGURE 5:**
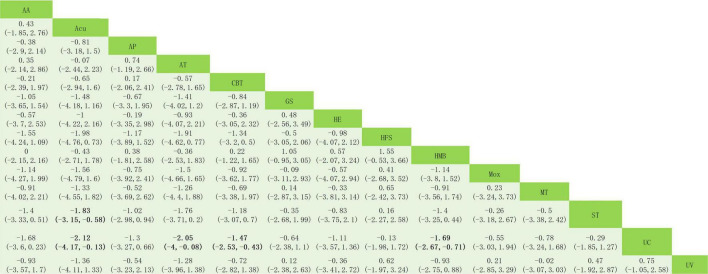
League table for paired comparison (SMD and 95% CI).

Effect estimates and 95% credible intervals for pairwise comparisons between different interventions are presented in Figures 4, 5. Compared with UC, significant improvements in pruritus symptoms were observed for Acupuncture (SMD = −2.12, 95% CI = −4.17 to −0.13), Aromatherapy (SMD = −2.05, 95% CI = −4.00 to −0.08), Combination Therapy (SMD = −1.47, 95% CI = −2.53 to −0.43), and Herbal Medicinal Bath (SMD = −1.69, 95% CI = −2.67 to −0.71). These differences were statistically significant. In contrast, Auricular Acupressure, Gua Sha, Herbal Enema, Herbal Fumigation and Steam, Acupressure, Moxibustion, Music Therapy, and Ultraviolet Phototherapy did not demonstrate statistical significance. Compared with Sham Treatment (ST), only Acupuncture (SMD = −1.83, 95% CI = −3.15 to −0.58) showed superior efficacy; no other comparisons reached statistical significance. No significant differences were observed between other paired comparisons for improving CKD-aP.

**FIGURE 6:**
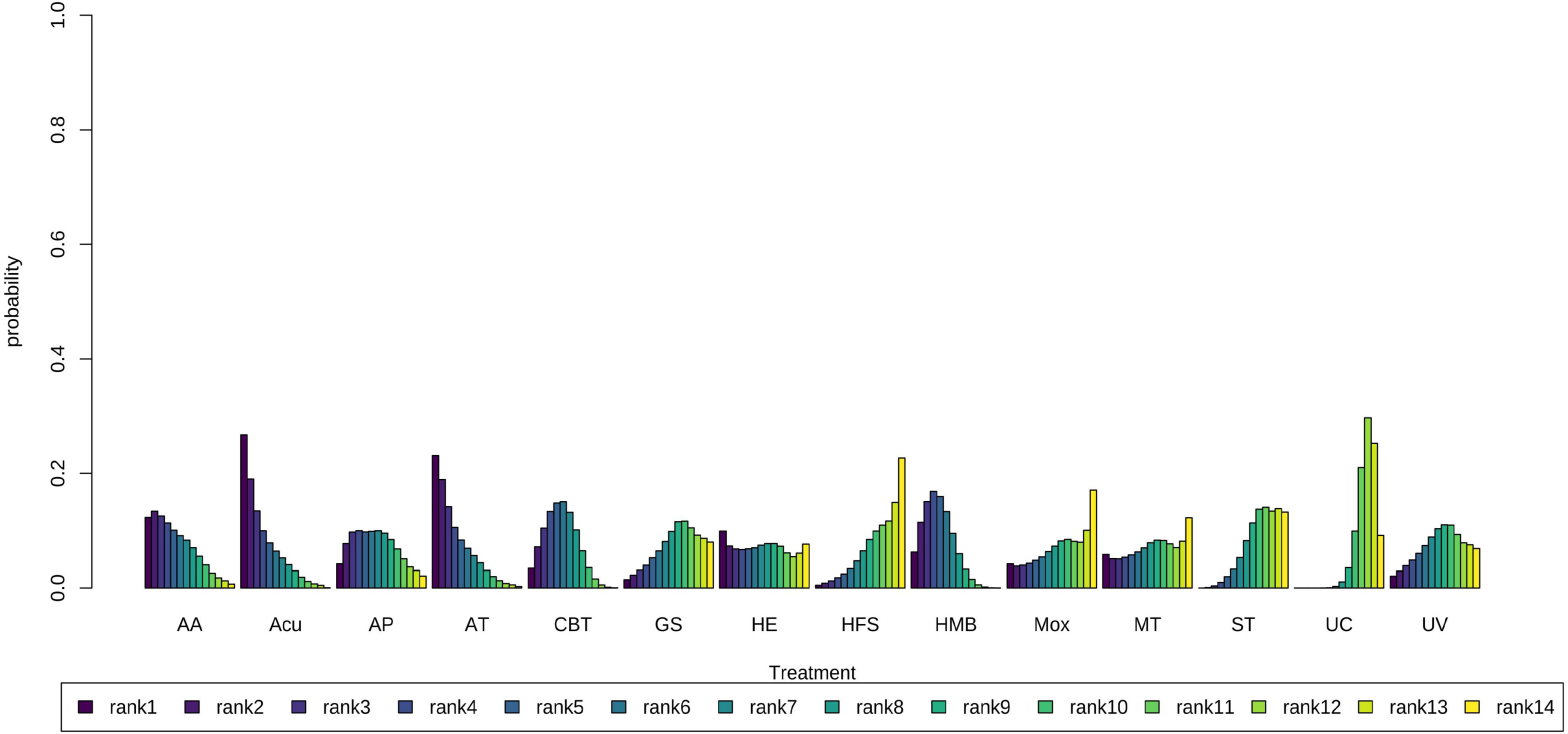
Ranking probability graph.

The ranking of interventions is presented in Figure 6. Acupuncture (Acu) was most likely to be the optimal treatment approach (SUCRA = 0.80), followed by Aromatherapy (AT) (SUCRA = 0.78) and Herbal Medicinal Bath (HMB) (SUCRA = 0.72).

### 3.4 Transitivity, heterogeneity, and inconsistency

Transitivity was assessed qualitatively using box plots to examine distributions of key clinical characteristics, including mean dialysis vintage, mean age, intervention duration, and the intensity of UC. The plots showed substantial overlap across most studies (see Supplementary Material 5), suggesting that potential effect modifiers were reasonably balanced across different interventions and that the transitivity assumption was generally tenable.

Overall heterogeneity was high, with τ^2^ = 1.35 (95% CI = 0.66–3.11) and I^2^ = 93.1% (95% CI = 86.8%–96.9%). Consequently, a random-effects model was selected for the analysis.

**FIGURE 7:**
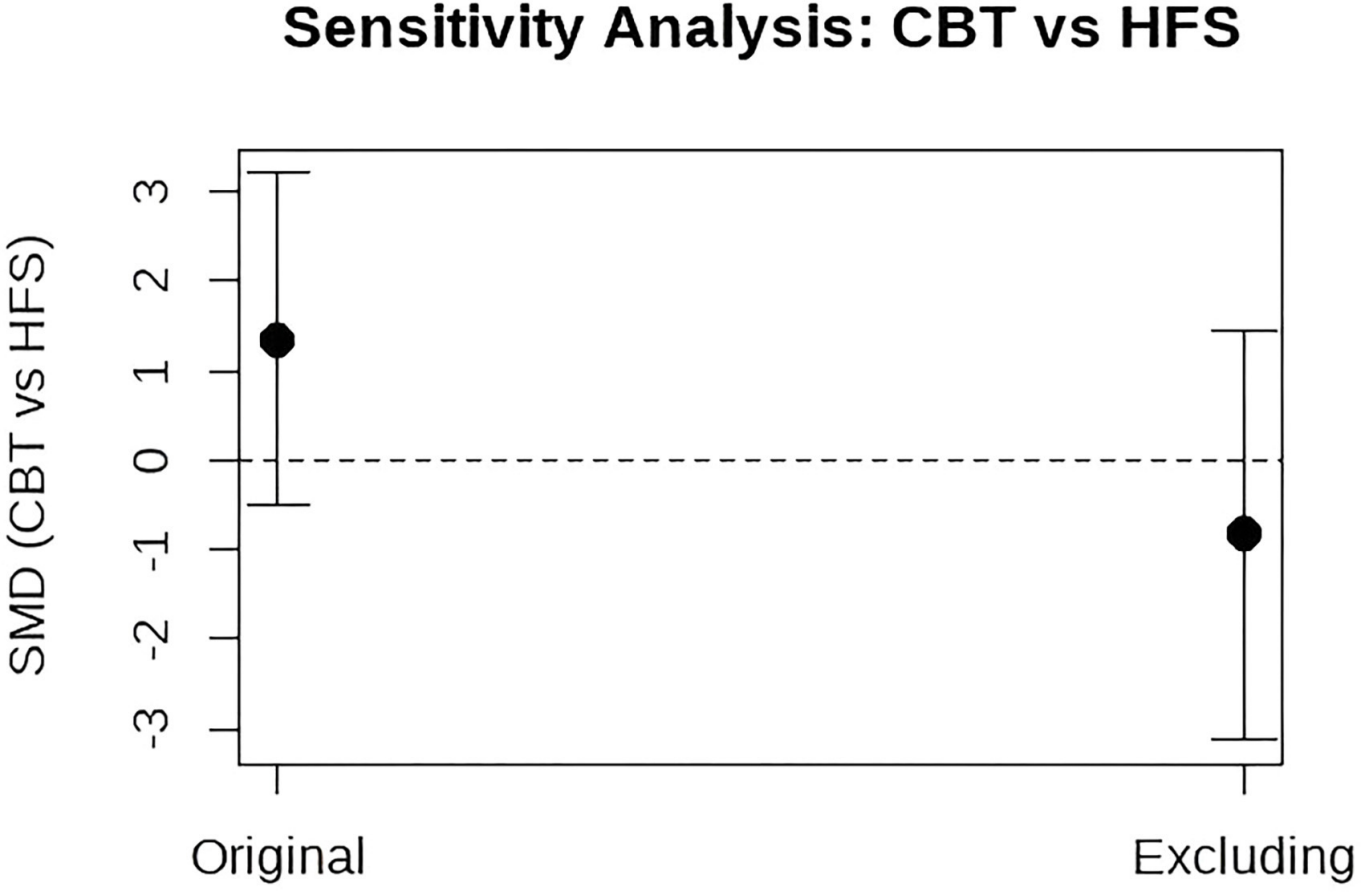
Effect size change for excluding direct comparison.

Global inconsistency testing revealed a difference in DIC of less than 1 between the consistency model (DIC = 61.9) and the inconsistency model (DIC = 62.7). This indicates no substantial global difference between the two models and suggests overall consistency between direct and indirect evidence. Therefore, the consistency model was retained. Local inconsistency testing using the node-splitting method showed that most direct and indirect comparisons were consistent (*P* > 0.05), except for the comparison between Combination Therapy (CBT) and Herbal Fumigation and Steam (HFS), which showed significant local inconsistency (*P* = 0.01). To explore the impact of this inconsistency, a sensitivity analysis was performed by excluding the study responsible for this direct comparison [Mao et al. (37)]. The results are presented in Figure 7. In this specific comparison, the direction of the pooled effect estimate changed, but the credible intervals under the two models partially overlapped. Treatment rankings exhibited some fluctuation: the SUCRA rank for HFS increased from 13th in the primary analysis to 3rd, while the rank for CBT decreased from 5th to 8th. These findings suggest that the result for this particular comparison is sensitive to the inclusion of a single study.

### 3.5 Meta-regression, subgroup analysis and sensitivity analysis

Given the high heterogeneity observed across studies, meta-regression was performed to explore potential sources of heterogeneity further. Among the five covariates examined, the 95% CIs for all regression coefficients crossed the null line (i.e., included zero), indicating that none of the included covariates had a statistically significant effect on the effect estimates. The DIC values for models incorporating these covariates were similar to that of the base model, and the heterogeneity parameters did not show a notable reduction. These findings confirm that the examined covariates were not the primary sources of the high heterogeneity observed in this study. Detailed results are provided in Supplementary Material 6.

We subsequently performed subgroup analyses. Because only one study included peritoneal dialysis patients, we were unable to stratify by dialysis modality (38). Moreover, the original studies lacked systematic reporting of relevant comorbidities and dialysis adequacy. We ultimately performed subgroup analyses stratified by pruritus assessment tool (VAS vs. non-VAS scales) and treatment duration (short-course [≤4 weeks] vs. long-course [>4 weeks]). The results showed that the τ-values across subgroups (ranging from 0.97 to 1.44) all fell within the 95% CI of the primary analysis (τ = 1.16, 95% CrI = 0.81–1.76), indicating that the assessment tool and treatment duration were not major contributors to the high heterogeneity. Full data provided in Supplementary Material 7.

Sensitivity analysis using the leave-one-out approach showed that after excluding any single study, the recalculated pooled SMDs ranged from −1.41 to −1.28. The 95% CIs for these estimates did not cross the null line, and the direction of the findings remained entirely consistent with the full model. The magnitude of change in effect estimates was small, with absolute differences ranging from 0.01 to 0.08. Although the heterogeneity parameter τ^2^ fluctuated slightly, it remained high overall, the results are consistent with the primary findings, indicating that the overall conclusions are robust and reliable. Detailed results are presented in Supplementary Material 8.

The sensitivity analysis that re-included the high-risk-of-bias studies showed that only HMB maintained a statistically significant difference, with an increased effect size (SMD = −2.40, 95% CI = −3.60 to −1.21), and rose to the top rank (SUCRA = 0.82). In contrast, the effect sizes of the other therapies, including Acu, all lost statistical significance. The heterogeneity parameter τ^2^ increased from 1.35 to 3.96. These findings indicate that although Acu ranked highly, its evidence base is relatively fragile, whereas the therapeutic effect of HMB is robust and supported by stable evidence. Moreover, the inclusion of high-risk studies further amplified between-study variability. Therefore, our conclusions should be interpreted with caution.

### 3.6 Publication bias and certainty of evidence assessment

**FIGURE 8:**
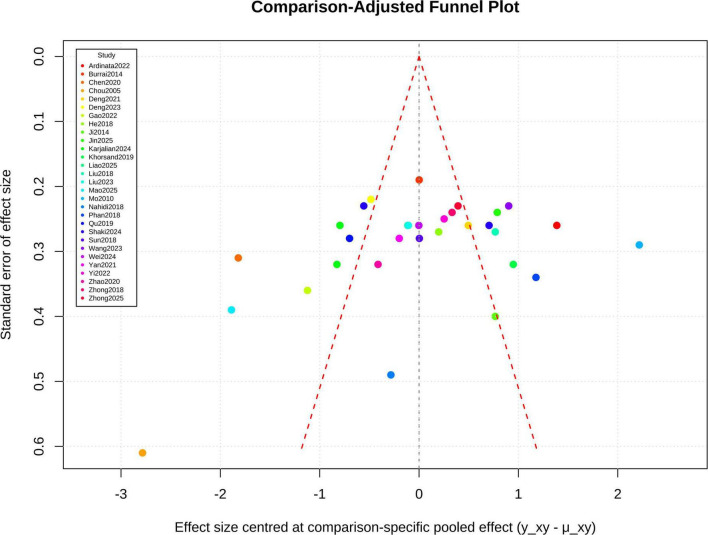
Funnel plot of comparison.

The comparison-adjusted funnel plot is presented in Figure 8, with the SMD plotted on the horizontal axis and the standard error on the vertical axis. Visual inspection suggests that the data points from the included studies are roughly symmetrically distributed. However, some studies fall outside the funnel boundaries, indicating the potential presence of small-study effects or publication bias in the included literature.

**FIGURE 9:**
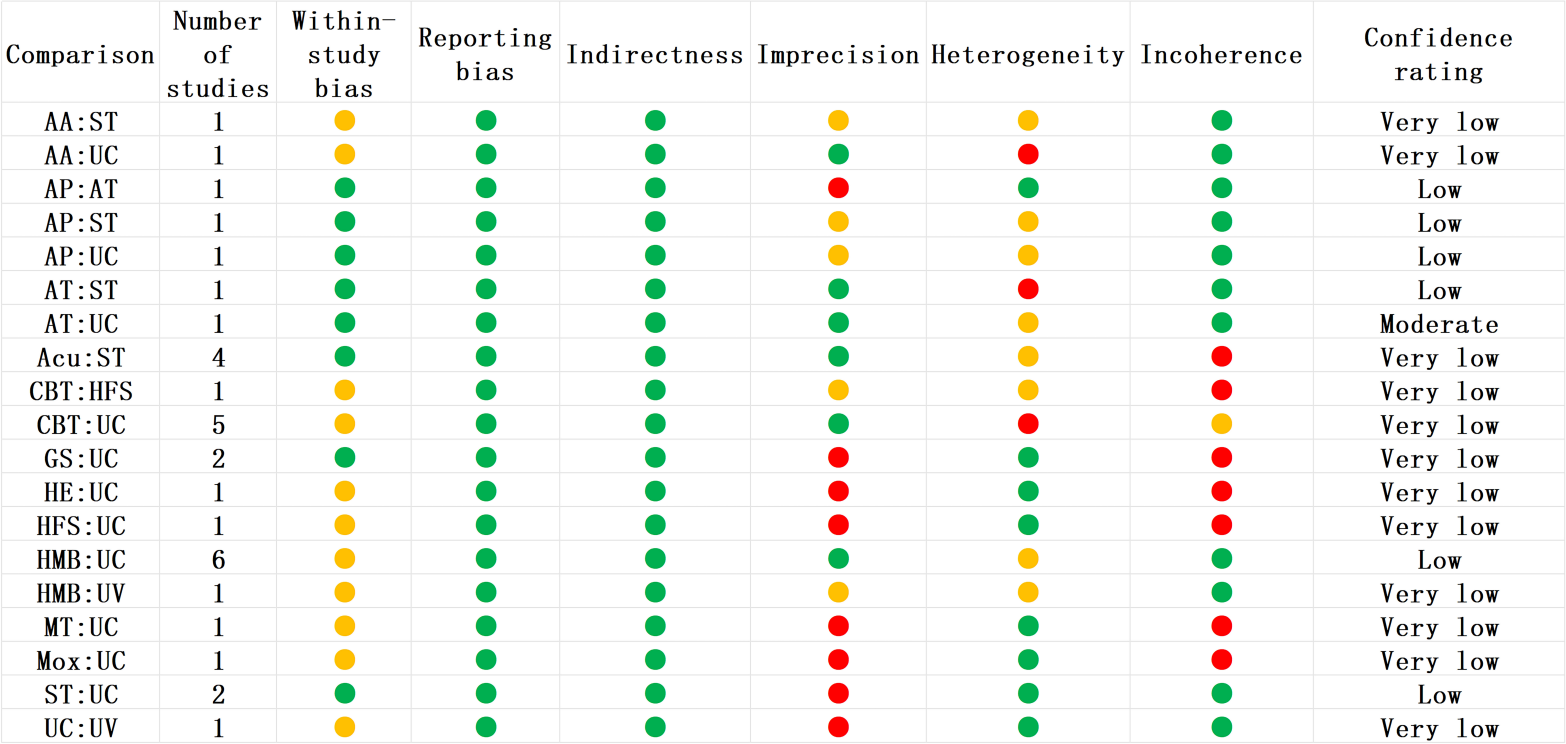
Summary table for credibility assessment. Risk of bias: 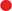
Major concerns or high. 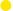
Some concerns or unclear. 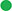
No concerns or low. The standardized minimum clinically important difference was set at 0.5.

The CINeMA results are presented in Figure 9. Except for AT, for which the certainty of evidence was rated as moderate, the certainty of evidence for all other interventions was rated as low or very low. Downgrading of the overall confidence in the pooled estimates was primarily attributable to within-study bias, imprecision, heterogeneity, and inconsistency.

### 3.7 Adverse events

Of the included studies, 18 (62%) did not report safety or adverse event information for the interventions. Among the 11 studies (38%) that reported adverse events, these events were observed only with Acu. Reported adverse events included pain, bleeding, and hematoma, and they occurred in both treatment and control groups. The incidence of pain ranged from 3.6% to 7.5%, and the incidence of bleeding ranged from 2.5% to 7.5%. Hematoma was reported in only one study (30), with an incidence of 1.85%. All adverse events were mild in severity and resolved spontaneously without affecting the study outcomes.

## 4 Discussion

This network meta-analysis represents the first comprehensive evaluation of the efficacy of major complementary therapies for chronic kidney disease-associated pruritus. In the primary analysis, we identified Acu as the preferred therapy. However, once low-quality evidence was incorporated, its treatment effect was no longer statistically significant. This instability, together with the sharp increase in the heterogeneity parameter, indicates that the evidence supporting the efficacy of Acu is not robust and is heavily dependent on a small number of high-quality studies. This empirical finding is fully consistent with the “very low” certainty rating assigned to Acu by the CINeMA assessment framework. In contrast, HMB demonstrated notable evidence stability. Its therapeutic effect remained consistently significant across both analysis scenarios, and the point estimate in the sensitivity analysis was not only stable but even increased. This suggests that the treatment effect of HMB is less sensitive to methodological bias, thereby offering greater confidence for clinical application. The therapeutic efficacy of HMB is believed to arise from synergistic interactions among physical, chemical, and mechanical factors. Warm water immersion itself can hydrate the stratum corneum, restore skin barrier function, and alleviate xerosis-associated pruritus (56). Concurrently, bioactive phytochemicals present in herbal decoctions or extracts can be absorbed through the skin, exerting local anti-inflammatory, antioxidant, and immunomodulatory effects (57, 58). A study by Suzuki et al. demonstrated that a fermented rice extract bath significantly reduced the VAS score for pruritus in hemodialysis patients, an effect potentially attributable to improved skin hydration and the elimination of mechanical friction during bathing (56). As a localized therapy delivered via the transdermal route, HMB offers significant safety advantages over oral or systemic treatment regimens. It does not compromise skin integrity, is generally well tolerated by patients, has a low incidence of adverse events, and does not impose additional metabolic burden on the kidneys. It is therefore particularly suitable for patients with chronic kidney disease (16).

In the analysis that excluded high-risk-of-bias studies, Acu, AT, and CBT also demonstrated efficacy superior to UC. Acu is a multi-target, multi-pathway intervention, and the precise mechanisms by which it alleviates CKD-aP have not yet been fully elucidated. However, existing evidence suggests that Acu may suppress the transmission of pruritic signals by modulating sensory nerve fiber conduction, exert antipruritic effects by regulating peripheral inflammatory mediators–including serum interleukin-2 (IL-2), histamine, and serotonin (5-HT)–and influence the release of endogenous opioid peptides while relieving pruritus through modulation of the balance between μ-opioid and κ-opioid receptors (59). Although Acu is an invasive procedure, local reactions are generally mild, and its overall safety profile is favorable, indicating its potential role in the management of CKD-aP. AT and CBT also showed certain advantages in relieving pruritus. The antipruritic effect of AT is likely mediated through both cutaneous and neuropsychological pathways. Studies have shown that essential oils such as lavender and peppermint possess significant anti-inflammatory and antioxidant properties, which can reduce the release of pruritogenic mediators–including histamine, serotonin, and substance P–from skin mast cells and sensory nerve endings (60, 61). Furthermore, aromatic compounds can act on the central nervous system via the olfactory pathway, modulating neurotransmitter systems such as serotonin and gamma-aminobutyric acid (GABA). The gentle massage frequently administered together with AT can also improve local microcirculation and skin hydration, thereby reducing pruritus and promoting relaxation. CBT, as represented in the included studies, involved the simultaneous or sequential application of two or more complementary therapies (e.g., herbal bath plus auricular acupressure, or herbal fumigation and steam plus acupressure). The enhanced efficacy of CBT may be attributed to synergistic or additive effects among its therapeutic components, each targeting distinct pathophysiological mechanisms. This multi-target synergy may produce a combined therapeutic effect that exceeds the sum of the individual parts. However, given the variability in combination regimens across studies, the specific synergistic mechanisms remain speculative, and further research is needed to identify optimal component combinations and their respective modes of action. It is noteworthy that, with the exception of AT, for which the certainty of evidence was rated as moderate, the evidence for all other therapies was rated as low to very low certainty, and the treatment effects varied depending on the quality of the included studies. This indicates considerable uncertainty regarding the current conclusions. Clinical decision-making should therefore be individualized according to patient circumstances and exercised with careful judgment based on the specific application context.

The direct and indirect evidence incorporated in this network meta-analysis were generally consistent, and the overall findings were reasonably robust. Although statistical inconsistency was detected for the comparison between CBT and HFS, and local sensitivity analysis revealed a directional change in the effect estimate and fluctuations in treatment rankings for this specific pair, the impact of this single study on the overall effect estimate was minimal and did not alter the core conclusions. This finding suggests that the evidence base linking these two specific interventions is relatively weak. The observed inconsistency may be attributable to chance arising from multiple comparisons, the limited number of studies providing direct evidence for this comparison, or differences in the specific intervention protocols involved. For instance, the CBT arm in the direct comparison study combined three distinct therapies (37), whereas other indirect comparisons involved combinations of only two. Therefore, this particular finding requires validation through future high-quality research.

Although the conclusions of this network meta-analysis are based on the best available evidence, interpretation should take into account the uncertainty introduced by high heterogeneity. We conducted meta-regression to explore five covariates: outcome measurement tool, treatment duration, sample size, publication year, and mean age. However, none of these factors adequately explained the high between-study heterogeneity. The subsequent subgroup analyses also showed that none of the stratification factors substantially reduced heterogeneity. This suggests that the observed heterogeneity may stem from more complex differences among the original studies, such as specific details of the intervention protocols (e.g., acupuncture point selection, herbal bath formulations), pruritus-related comorbidities, dialysis adequacy, and baseline pruritus severity. Sensitivity analysis also indicated that heterogeneity likely stems from the combined influence of multiple studies rather than a single outlier, the overall findings were not unduly influenced by any single study.

High heterogeneity indicates substantial variability across studies, which reduces the precision and certainty of the pooled effect estimates. Therefore, the treatment rankings should be interpreted with great caution. Although HMB demonstrated robust efficacy, the overall evidence remains insufficient to support strong clinical practice recommendations. While we cannot provide a definitive answer regarding the optimal treatment, our findings can indicate which therapies merit further investigation. Clinicians should consider these results as a provisional reference and integrate them with patient preferences, local resource availability, and individual risk–benefit assessments. The high heterogeneity also underscores the urgent need for future research to develop standardized intervention protocols, so as to achieve more precise and comparable effect estimates.

Overall, the included studies demonstrated similarity across study populations, intervention categories, control conditions, and outcome measures. A qualitative assessment of transitivity was also performed for key clinical characteristics. The negative results of the meta-regression suggest that the measured factors did not act as effect modifiers. Furthermore, box plots confirmed that key clinical characteristics were reasonably balanced across the various comparison groups. Consequently, the transitivity assumption was generally supported, and the evidence possesses a reasonable degree of external validity.

## 5 Strengths and limitations

This systematic review and network meta-analysis provides a comprehensive synthesis of data regarding the efficacy and safety of established complementary therapies for alleviating chronic kidney disease-associated pruritus and offers recommendations for optimal clinical approaches based on the analytical findings. All included studies were randomized controlled trials; those with an overall high risk of bias were excluded, and the evidence underwent rigorous quality assessment. Consequently, the evidence presented here is more robust than that of previous reviews. Nonetheless, several limitations should be acknowledged: (1) the sensitivity analysis showed that including high-risk-of-bias studies introduced substantial heterogeneity and altered the treatment rankings. On one hand, this validates the rationale for excluding high-risk-of-bias studies from the primary analysis; on the other hand, it highlights the fragility of the primary findings. (2) Certain comparisons were based on single studies with small sample sizes. Moreover, the diversity of complementary therapy modalities and variations in their implementation may have influenced the precision of effect estimates and the certainty of evidence. (3) The search was restricted to studies published in English or Chinese, which may introduce language bias. (4) Most trials were conducted in China, potentially limiting the cross-cultural applicability of the findings. (5) Given the substantial heterogeneity among the included studies, the generalizability of the results may be constrained. (6) Inadequate reporting of adverse events in the majority of the original studies limits the assessment of the risk–benefit profile of the interventions. (7) Comorbidities were not systematically reported in the included studies, making it difficult to conduct meta-regression or subgroup analyses based on these factors, therefore, their impact on pruritus could not be assessed.

## 6 Conclusion

Based on the current evidence, HMB demonstrates a robust and significant therapeutic profile and is tentatively recommended as the more reliable adjunctive treatment for alleviating CKD-aP. In contrast, the treatment effect of Acu is highly sensitive to study quality, and the existing evidence base is insufficient to support a strong clinical recommendation. Acu can be regarded as a potential alternative that requires validation through high-quality studies. Given the limited number of included studies, the presence of some risk of bias, the low certainty of evidence, and the high heterogeneity, these conclusions should be interpreted with caution. Future research should prioritize the following areas: (1) conducting multicenter, double-blind randomized controlled trials with larger sample sizes; (2) further exploring the mechanisms of action and standardized intervention protocols for Acu and HMB (e.g., acupoint selection, herbal bath formulations); (3) systematically documenting adverse events and comorbidities that may influence pruritus; and (4) conducting cross-regional studies to assess the generalizability of the findings.

## Data Availability

The original contributions presented in this study are included in this article/Supplementary material, further inquiries can be directed to the corresponding author.
