# Supplementary material for: Effects of different complementary therapies on chronic kidney disease-associated pruritus: a systematic review and network meta-analysis
Source: Front Med (Lausanne). 2026 Jul 22;13:1860437. doi: 10.3389/fmed.2026.1860437 (PMC13437412; doi:10.3389/fmed.2026.1860437)
Supplement: Supplementary file 1 [file Data_Sheet_1.zip › Appendix A Supplementary materials/Supplementary material 3-PSRF.PDF]

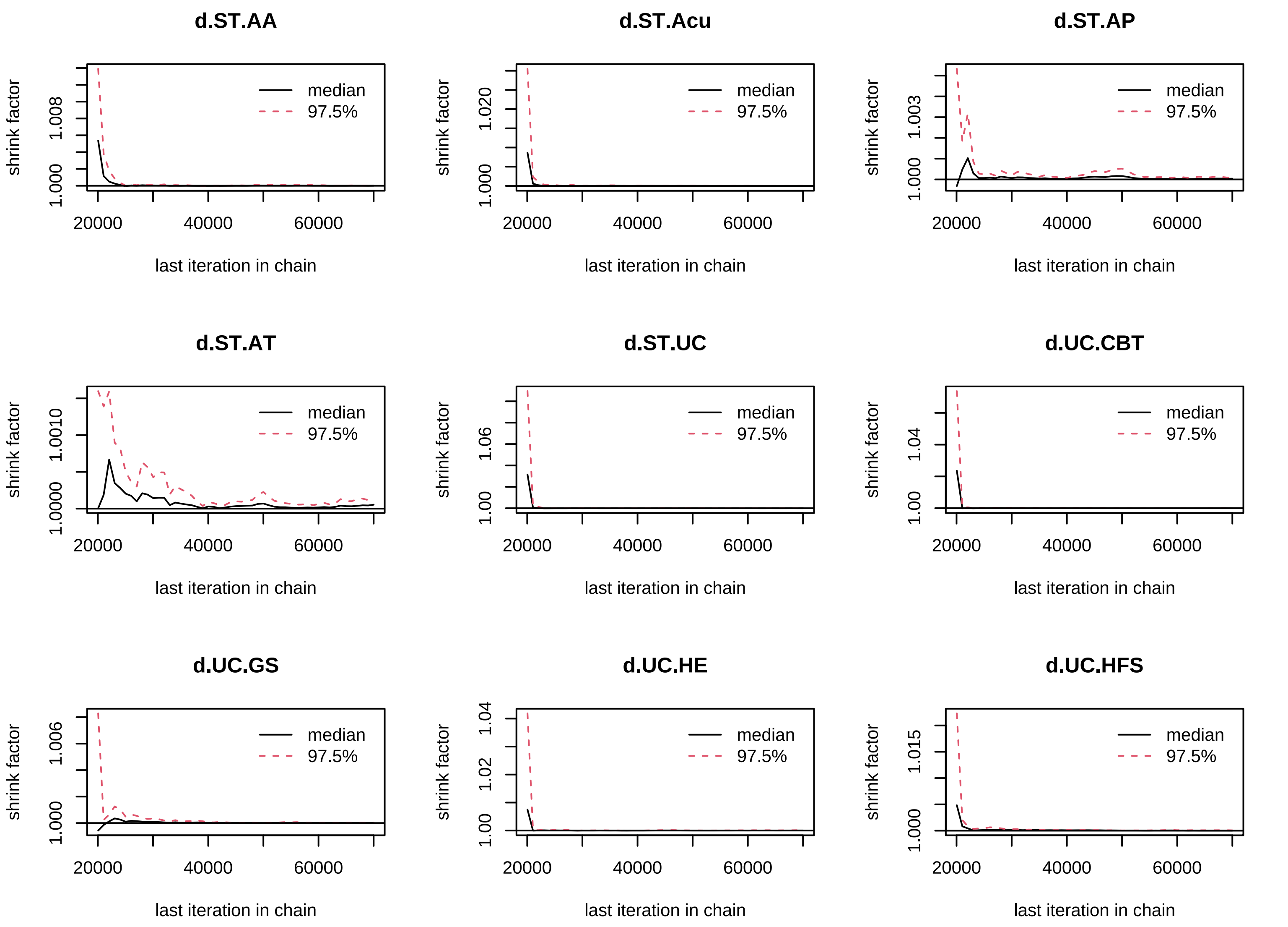
d.ST.AA
shrink factor
1.000
1.008
median
97.5%
20000
40000
60000
last iteration in chain
d.ST.Acu
shrink factor
1.000
1.020
median
97.5%
20000
40000
60000
last iteration in chain
d.ST.AP
shrink factor
1.000
1.003
median
97.5%
20000
40000
60000
last iteration in chain
d.ST.AT
shrink factor
1.0000
1.0010
median
97.5%
20000
40000
60000
last iteration in chain
d.ST.UC
shrink factor
1.00
1.06
median
97.5%
20000
40000
60000
last iteration in chain
d.UC.CBT
shrink factor
1.00
1.04
median
97.5%
20000
40000
60000
last iteration in chain
d.UC.GS
shrink factor
1.000
1.006
median
97.5%
20000
40000
60000
last iteration in chain
d.UC.HE
shrink factor
1.00
1.02
1.04
median
97.5%
20000
40000
60000
last iteration in chain
d.UC.HFS
shrink factor
1.000
1.015
median
97.5%
20000
40000
60000
last iteration in chain

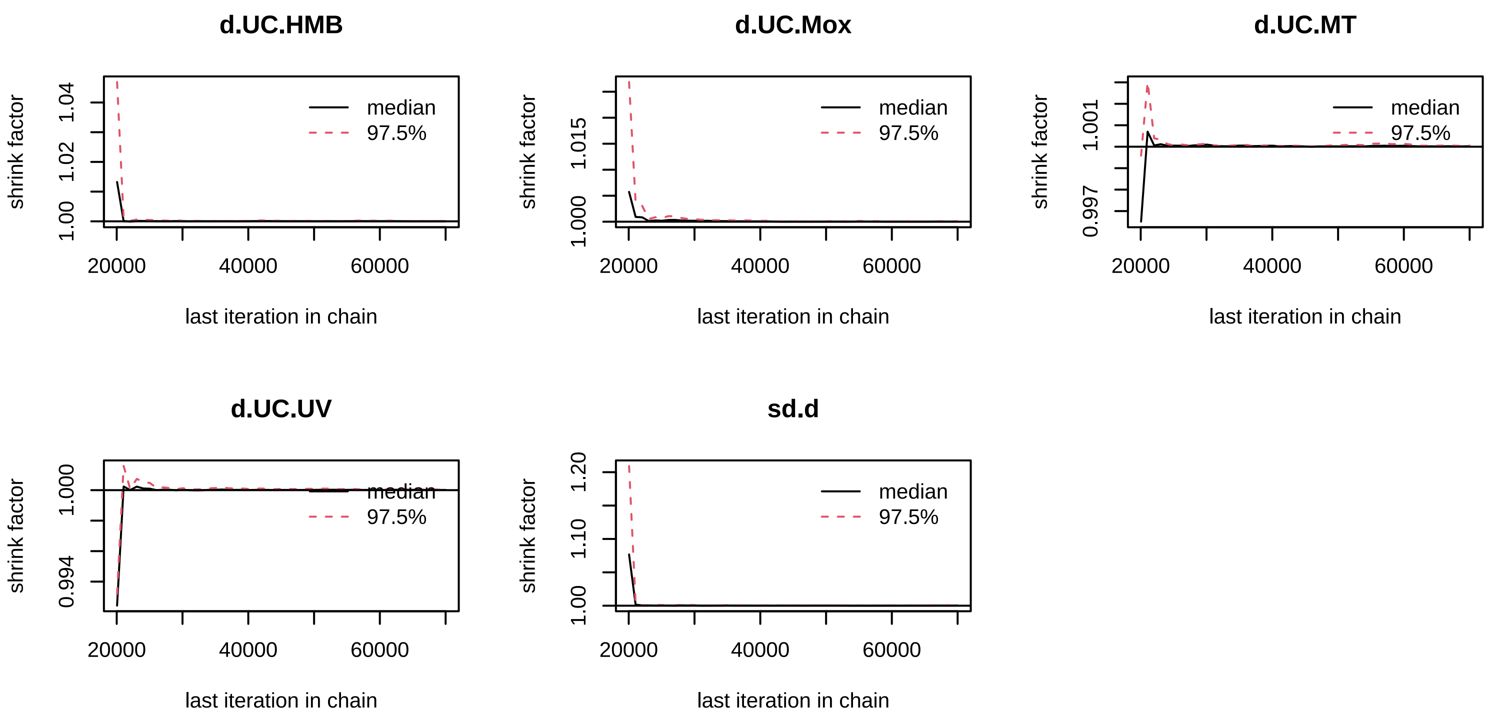
d.UC.HMB
d.UC.Mox
d.UC.MT
d.UC.UV
sd.d
shrink factor
last iteration in chain
median
97.5%
20000
40000
60000
1.00
1.02
1.04
1.000
1.015
0.997
1.001
0.994
1.000
1.00
1.10
1.20
