# Supplementary material for: Effects of different complementary therapies on chronic kidney disease-associated pruritus: a systematic review and network meta-analysis
Source: Front Med (Lausanne). 2026 Jul 22;13:1860437. doi: 10.3389/fmed.2026.1860437 (PMC13437412; doi:10.3389/fmed.2026.1860437)
Supplement: Supplementary file 1 [file Data_Sheet_1.zip › Appendix A Supplementary materials/Supplementary material 4-Trace and Density Plot.PDF]

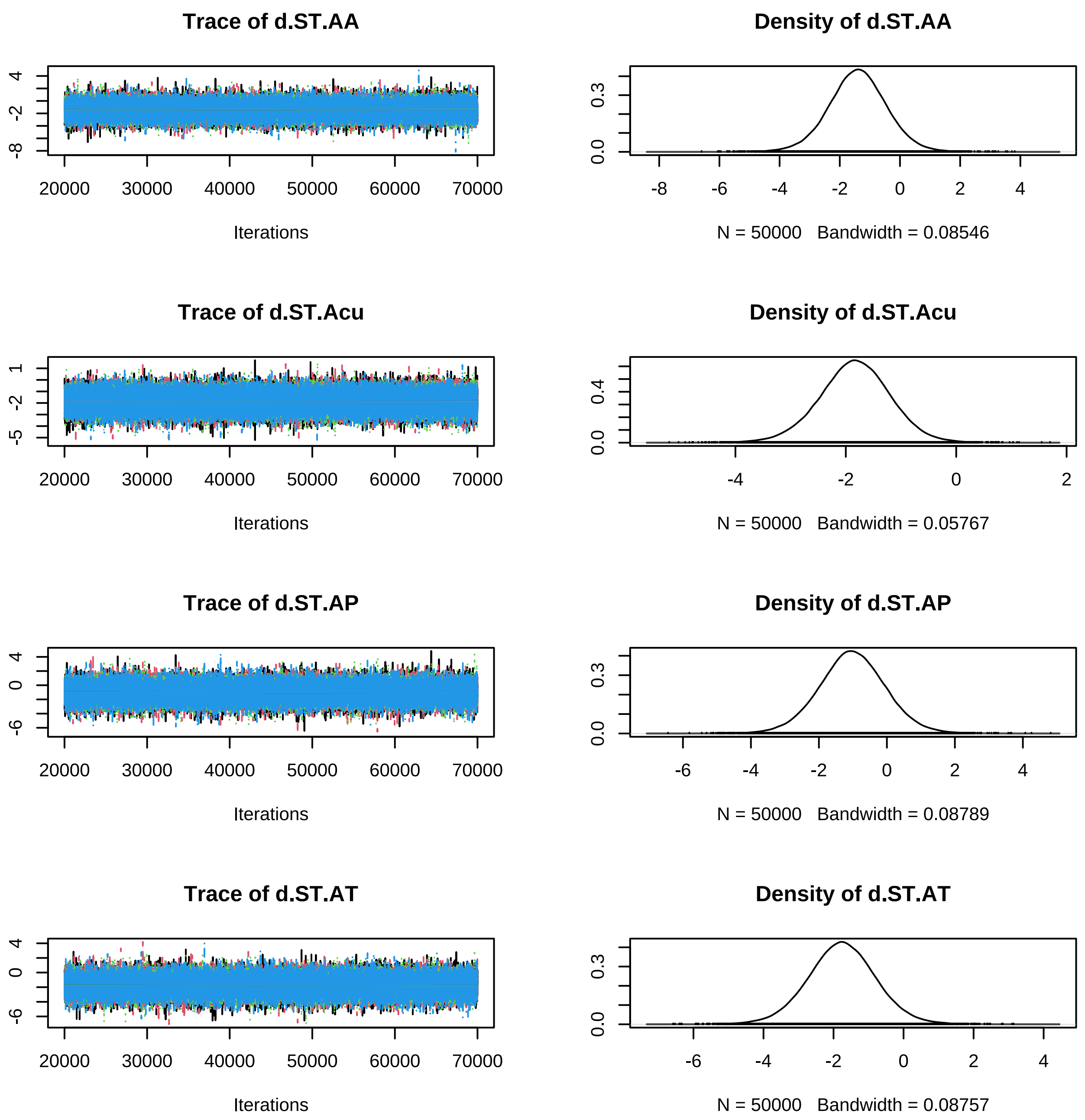
Trace of d.ST.AA
Iterations
Density of d.ST.AA
N = 50000 Bandwidth = 0.08546
Trace of d.ST.Acu
Iterations
Density of d.ST.Acu
N = 50000 Bandwidth = 0.05767
Trace of d.ST.AP
Iterations
Density of d.ST.AP
N = 50000 Bandwidth = 0.08789
Trace of d.ST.AT
Iterations
Density of d.ST.AT
N = 50000 Bandwidth = 0.08757

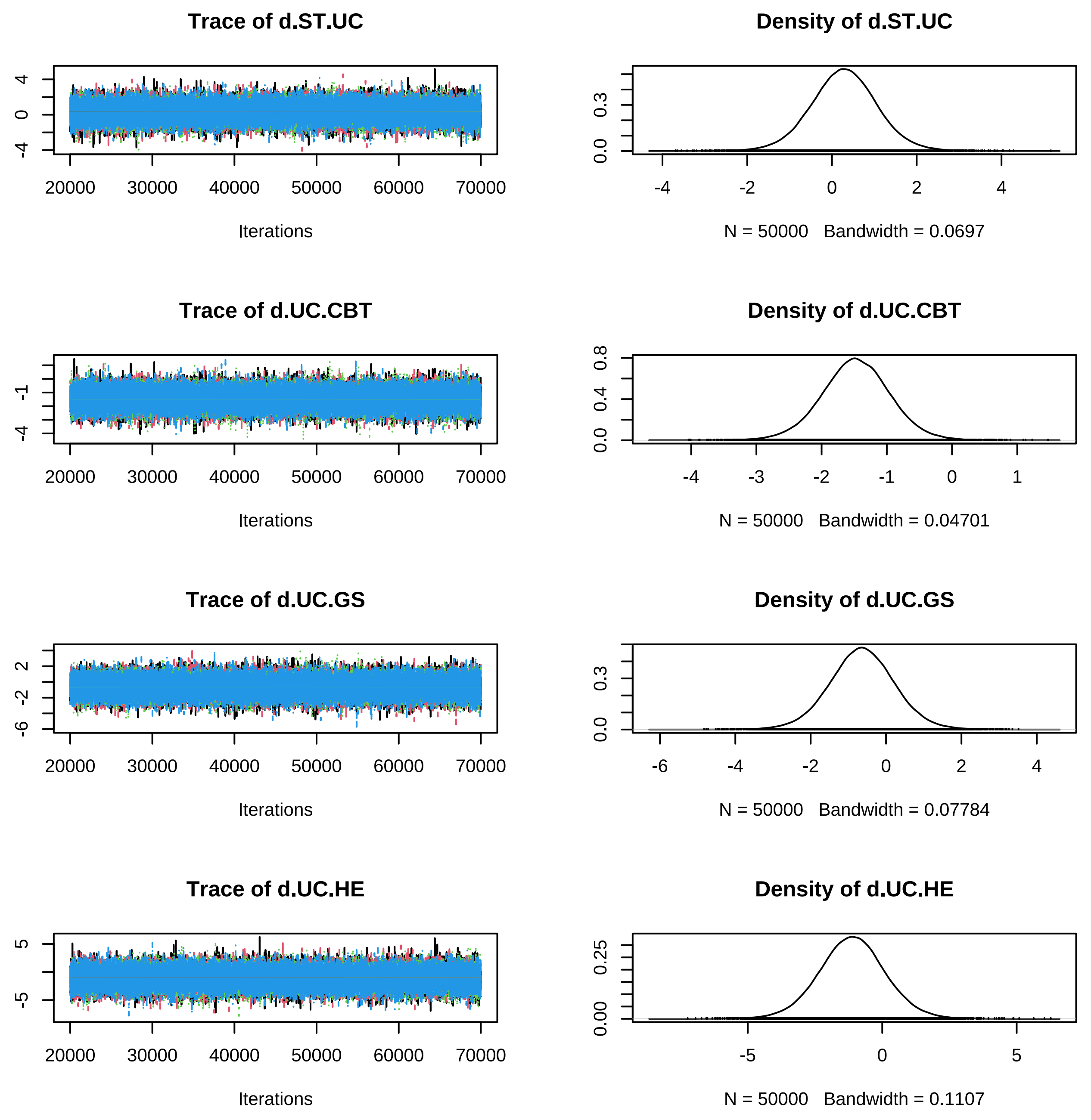
Trace of d.ST.UC
Iterations
Density of d.ST.UC
N = 50000 Bandwidth = 0.0697
Trace of d.UC.CBT
Iterations
Density of d.UC.CBT
N = 50000 Bandwidth = 0.04701
Trace of d.UC.GS
Iterations
Density of d.UC.GS
N = 50000 Bandwidth = 0.07784
Trace of d.UC.HE
Iterations
Density of d.UC.HE
N = 50000 Bandwidth = 0.1107

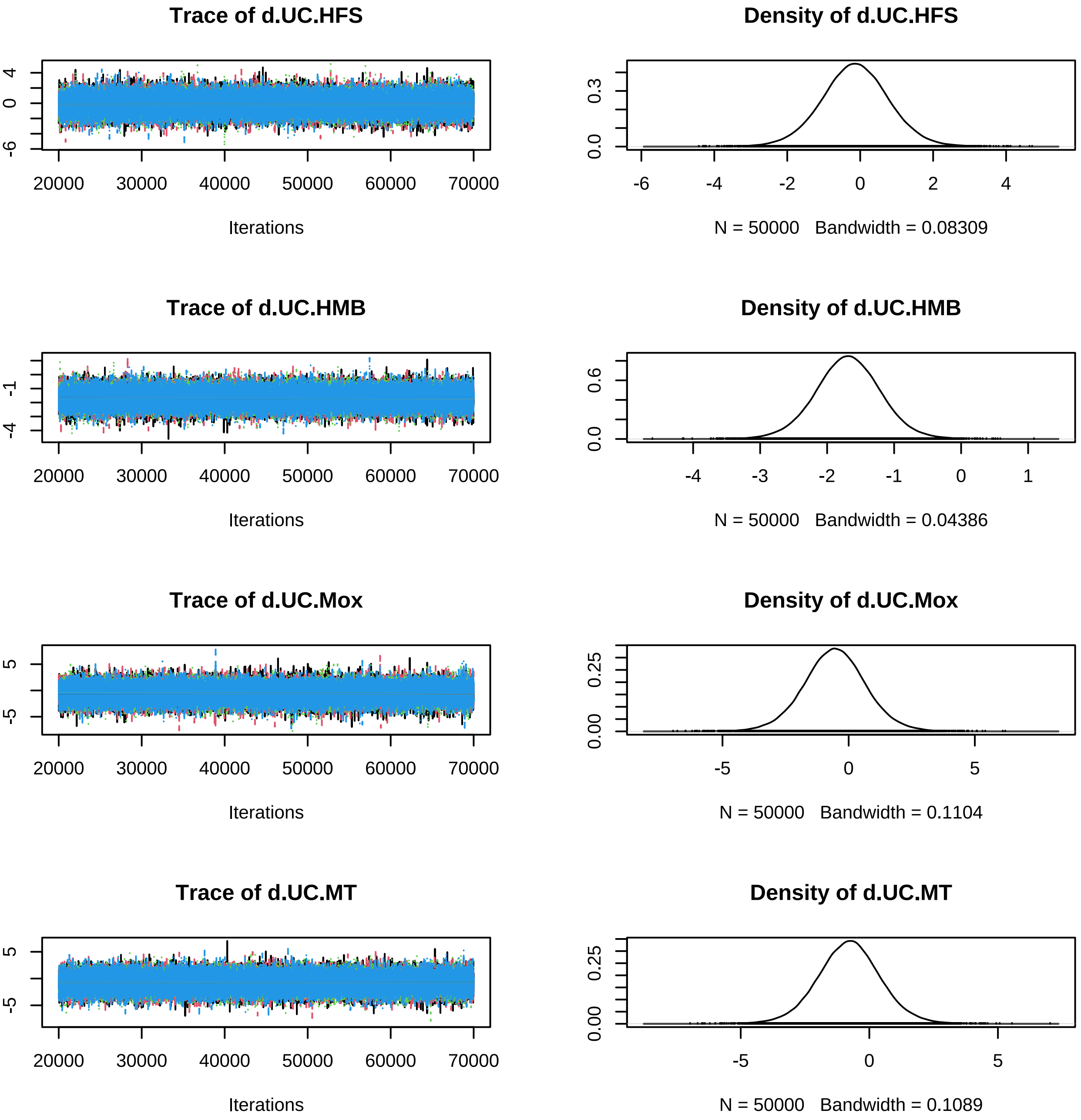
Trace of d.UC.HFS
Iterations
Density of d.UC.HFS
N = 50000 Bandwidth = 0.08309
Trace of d.UC.HMB
Iterations
Density of d.UC.HMB
N = 50000 Bandwidth = 0.04386
Trace of d.UC.Mox
Iterations
Density of d.UC.Mox
N = 50000 Bandwidth = 0.1104
Trace of d.UC.MT
Iterations
Density of d.UC.MT
N = 50000 Bandwidth = 0.1089

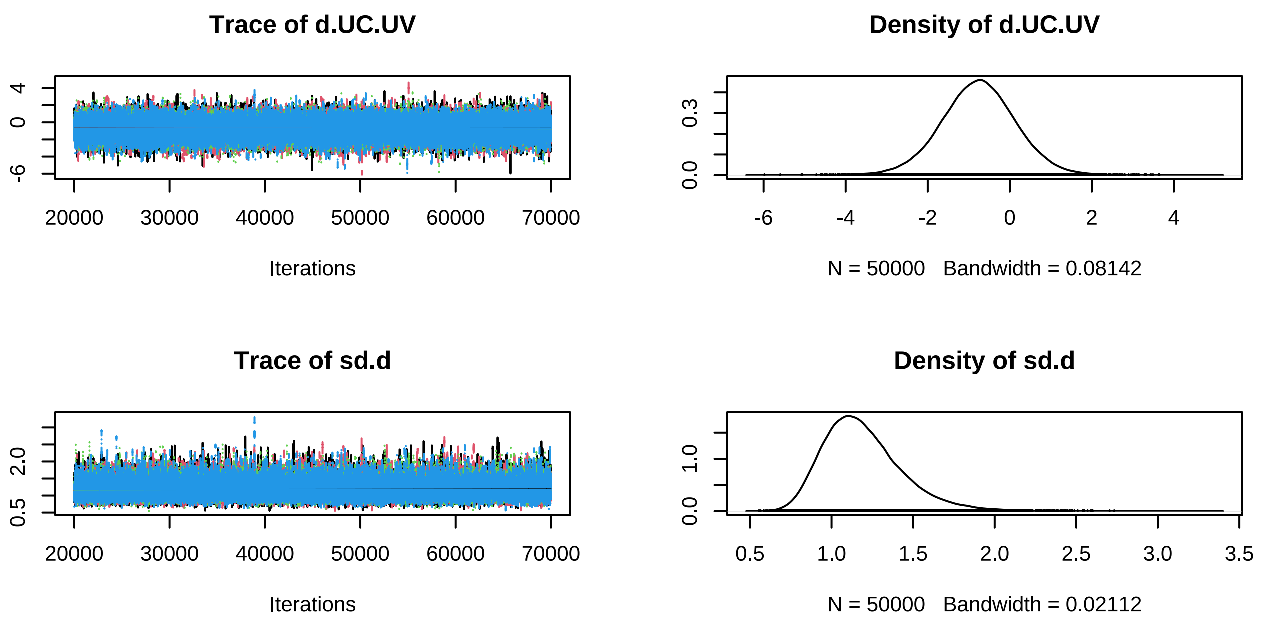
Trace of d.UC.UV
Iterations
Density of d.UC.UV
N = 50000 Bandwidth = 0.08142
Trace of sd.d
Iterations
Density of sd.d
N = 50000 Bandwidth = 0.02112
