# Supplementary material for: Effects of different complementary therapies on chronic kidney disease-associated pruritus: a systematic review and network meta-analysis
Source: Front Med (Lausanne). 2026 Jul 22;13:1860437. doi: 10.3389/fmed.2026.1860437 (PMC13437412; doi:10.3389/fmed.2026.1860437)
Supplement: Supplementary file 1 [file Data_Sheet_1.zip › Appendix A Supplementary materials/Supplementary material 7-Subgroup analysis.docx]

**Table 1. Summary of Heterogeneity Parameters Across Subgroup Analyses**

| Subgroup | Number of Included Studies | τ(95% CI) | τ² | Heterogeneity Explained |
| --- | --- | --- | --- | --- |
| Primary analysis | 29 | 1.16 (0.81–1.76) | 1.35 | Reference |
| VAS scale | 10 | 1.44 (0.57–2.70) | 2.07 | No |
| Non-VAS scale | 19 | 1.37 (0.87–2.40) | 1.87 | No |
| Short-course (≤4 w) | 18 | 1.24 (0.77–2.34) | 1.54 | No |
| Long-course (>4 w) \| | 11 | 0.97 (0.45–2.29) | 0.95 | No (CI overlapped) |

Abbreviations:

CI: credible interval; VAS: visual analog scale; τ: between-study standard deviation (heterogeneity parameter); τ²: between-study variance.
