# Supplementary material for: Effects of different complementary therapies on chronic kidney disease-associated pruritus: a systematic review and network meta-analysis
Source: Front Med (Lausanne). 2026 Jul 22;13:1860437. doi: 10.3389/fmed.2026.1860437 (PMC13437412; doi:10.3389/fmed.2026.1860437)
Supplement: Supplementary file 1 [file Data_Sheet_1.zip › Appendix A Supplementary materials/Supplementary material 8-Sensitivity analysis.pdf]

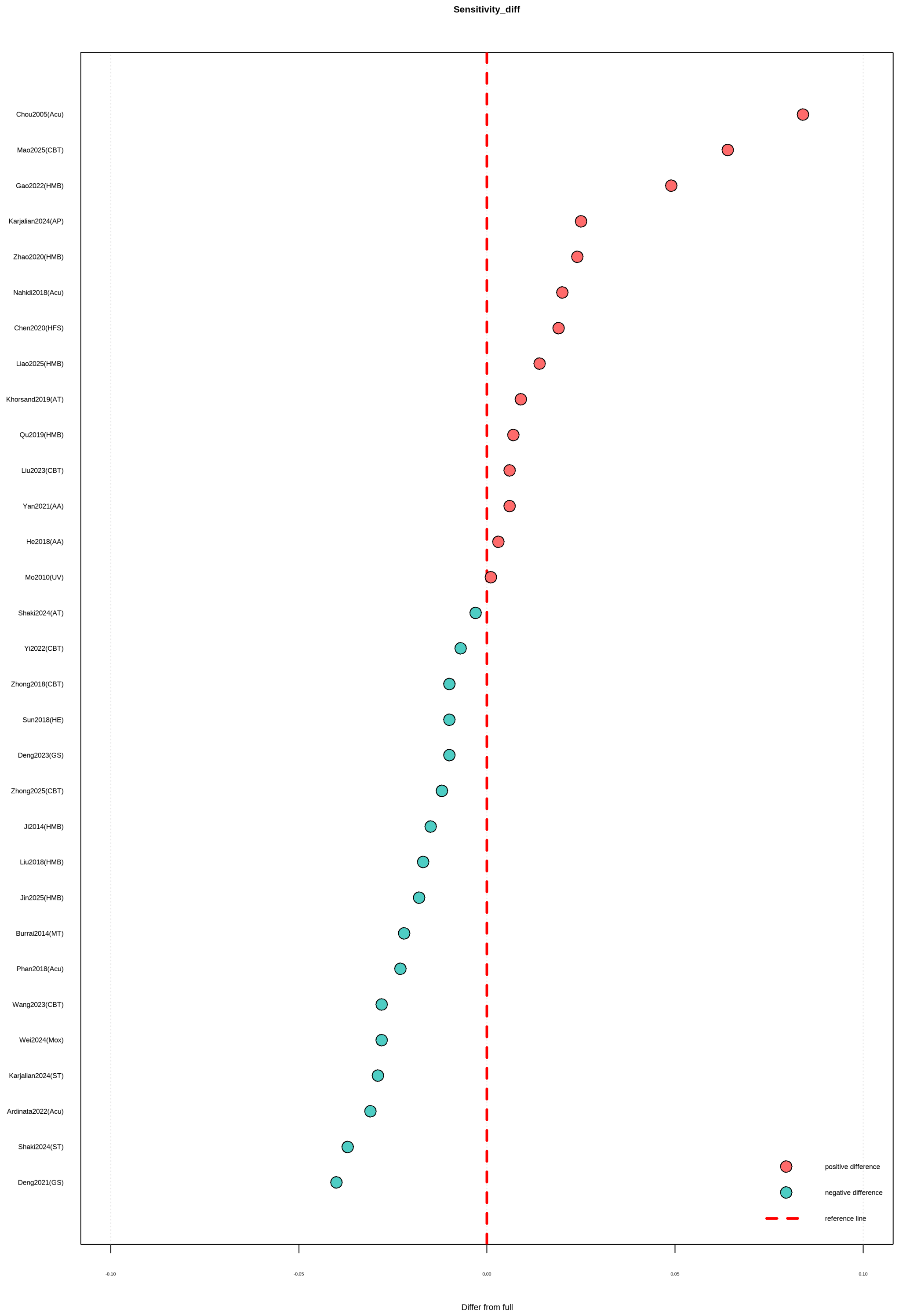

Sensitivity_diff
Chou2005(Acu)
Mao2025(CBT)
Gao2022(HMB)
Karjalian2024(AP)
Zhao2020(HMB)
Nahidi2018(Acu)
Chen2020(HFS)
Liao2025(HMB)
Khorsand2019(AT)
Qu2019(HMB)
Liu2023(CBT)
Yan2021(AA)
He2018(AA)
Mo2010(UV)
Shaki2024(AT)
Yi2022(CBT)
Zhong2018(CBT)
Sun2018(HE)
Deng2023(GS)
Zhong2025(CBT)
Ji2014(HMB)
Liu2018(HMB)
Jin2025(HMB)
Burrai2014(MT)
Phan2018(Acu)
Wang2023(CBT)
Wei2024(Mox)
Karjalian2024(ST)
Ardinata2022(Acu)
Shaki2024(ST)
Deng2021(GS)
positive difference
negative difference
reference line
-0.10
-0.05
0.00
0.05
0.10
Differ from full

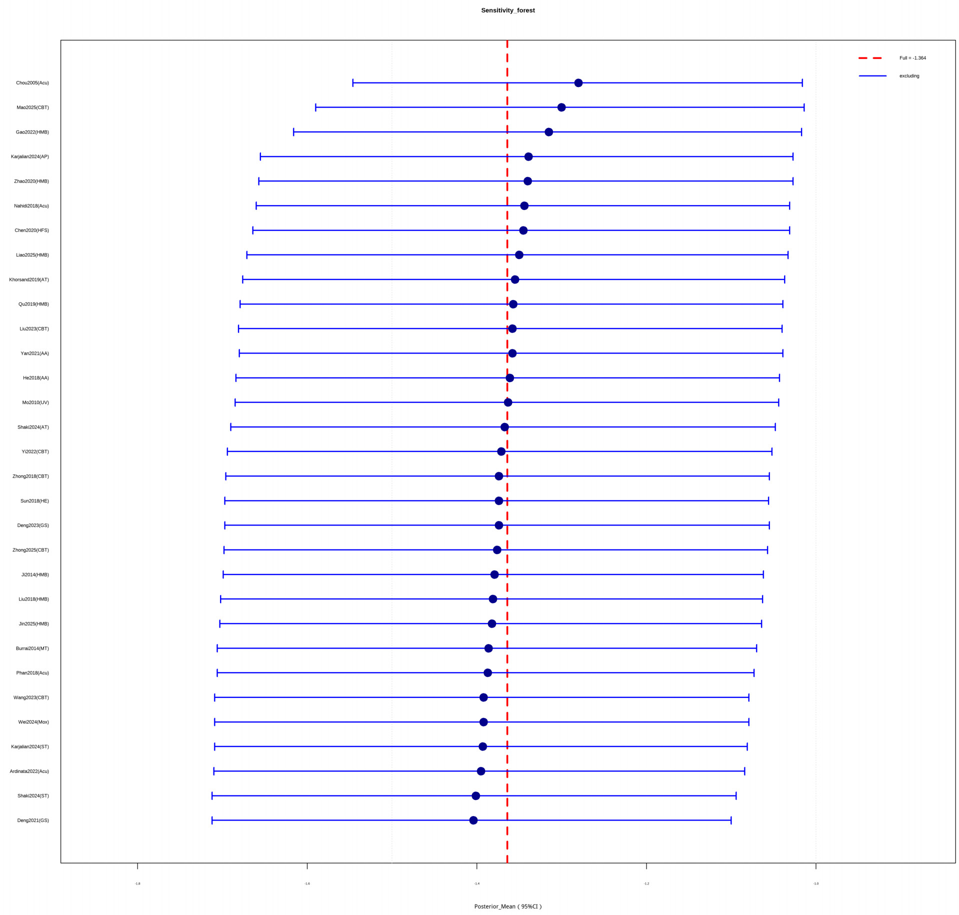
Sensitivity_forest
Full = -1.364
excluding
Chou2005(Acu)
Mao2025(CBT)
Gao2022(HMB)
Karjalian2024(AP)
Zhao2020(HMB)
Nahidi2018(Acu)
Chen2020(HFS)
Liao2025(HMB)
Khorsand2019(AT)
Qu2019(HMB)
Liu2023(CBT)
Yan2021(AA)
He2018(AA)
Mo2010(UV)
Shaki2024(AT)
Yi2022(CBT)
Zhong2018(CBT)
Sun2018(HE)
Deng2023(GS)
Zhong2025(CBT)
Ji2014(HMB)
Liu2018(HMB)
Jin2025(HMB)
Burrai2014(MT)
Phan2018(Acu)
Wang2023(CBT)
Wei2024(Mox)
Karjalian2024(ST)
Ardinata2022(Acu)
Shaki2024(ST)
Deng2021(GS)
-1.8
-1.6
-1.4
-1.2
-1.0
Posterior_Mean ( 95%CI )

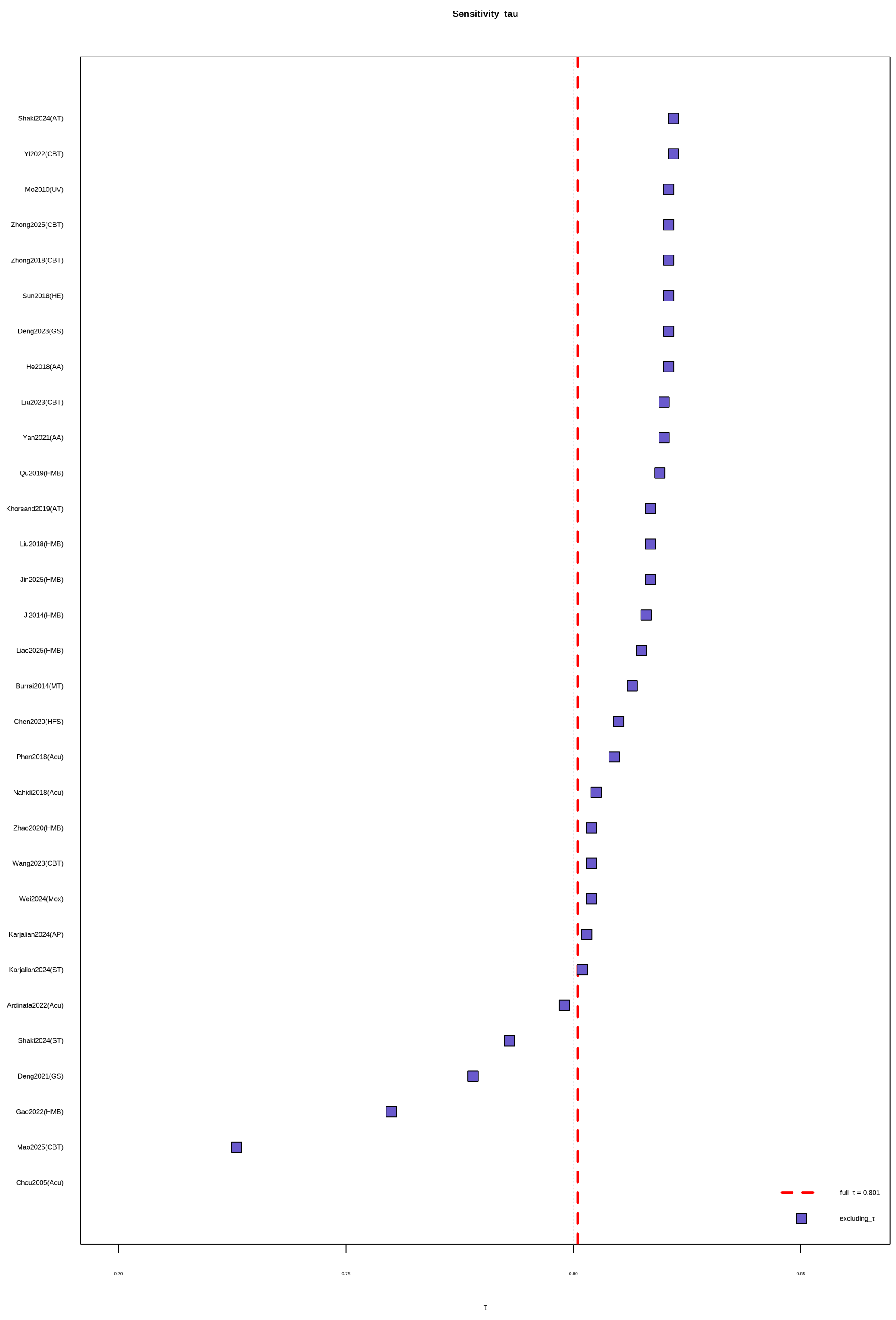
Sensitivity_tau
Shaki2024(AT)
Yi2022(CBT)
Mo2010(UV)
Zhong2025(CBT)
Zhong2018(CBT)
Sun2018(HE)
Deng2023(GS)
He2018(AA)
Liu2023(CBT)
Yan2021(AA)
Qu2019(HMB)
Khorsand2019(AT)
Liu2018(HMB)
Jin2025(HMB)
Ji2014(HMB)
Liao2025(HMB)
Burrai2014(MT)
Chen2020(HFS)
Phan2018(Acu)
Nahidi2018(Acu)
Zhao2020(HMB)
Wang2023(CBT)
Wei2024(Mox)
Karjalian2024(AP)
Karjalian2024(ST)
Ardinata2022(Acu)
Shaki2024(ST)
Deng2021(GS)
Gao2022(HMB)
Mao2025(CBT)
Chou2005(Acu)
full_τ = 0.801
excluding_τ
0.70
0.75
0.80
0.85
τ
